# The Effectiveness of Polynucleotides in Esthetic Medicine: A Systematic Review

**DOI:** 10.1111/jocd.16721

**Published:** 2024-12-08

**Authors:** Smaragda Lampridou, Sian Bassett, Maurizio Cavallini, George Christopoulos

**Affiliations:** ^1^ Department of Surgery and Cancer Imperial College London London UK; ^2^ Sian Aesthetics, Highgate London UK; ^3^ Dermatology and Dermatologic Surgery Centro Diagnostico Italiano Milan Italy; ^4^ The Ghanem Clinic London UK; ^5^ College of Medicine and Dentistry Ulster University Coleraine UK

**Keywords:** esthetic medicine, polydeoxyribonucleotide (PDRN), polynucleotide (PN), systematic review

## Abstract

**Background:**

Polynucleotides (PN), popular in biorevitalization, show promise in the current sphere of esthetic medicine due to their regenerative properties, previously used in wound healing. Currently, research investigates their use in esthetic medicine. The aim of this review was to synthesize the existing literature, focusing on the effectiveness of PN in esthetic medicine, which is concentrated on skin rejuvenation by providing patients with multiple benefits and the least side effects.

**Methods:**

A systematic electronic search was conducted in Embase, Medline, and Cochrane to identify primary research studies evaluating the effectiveness of polynucleotides in esthetic medicine, published in English between January 01, 2010, and January 01, 2024. A narrative synthesis was reported according to the Preferred Reporting Items for Systematic Reviews and Meta‐Analyses statement. The quality of evidence was assessed using the Critical Appraisal Skills Programme checklists. PROSPERO Registration: CRD42024588712.

**Results:**

Nine studies, of low and moderate quality, were included in this review, describing a population of 219 patients receiving PN treatment. A variation was present regarding procedural characteristics, such as injection areas and techniques. Polynucleotide injections have shown promising outcomes in reducing wrinkles, improving skin texture, and enhancing elasticity, with statistically significant results in several studies. While side effects are generally mild and transient, patient satisfaction is moderate to high, suggesting PN treatment as a well‐tolerated and effective cosmetic intervention.

**Conclusion:**

Polynucleotides offer promising potential in esthetic medicine; however, there is limited consensus regarding their optimal use. Rigorous, high‐quality studies are essential to validate the effectiveness and safety of PN.

## 1 Introduction

In the rapidly evolving landscape of medical esthetics, skin rejuvenation has become a key trend, encompassing multiple techniques to enhance skin health, restore elasticity, refine texture, and achieve a youthful appearance [1]. Popular treatments, including laser skin resurfacing, microdermabrasion, chemical peels, microneedling, dermal fillers, botulinum toxin, and intense pulsed light, aim to boost collagen production, remove dead skin layers, encourage cell renewal, and address wrinkles or skin irregularities for smoother, revitalized skin [2, 3]. Increasing demand for natural, minimally invasive, and scientifically validated solutions has further driven interest in effective and low‐risk dermatological interventions that ensure a swift recovery, allowing patients to seamlessly resume their social lives.

Polynucleotide‐based biorevitalization, particularly using polynucleotides (PN) and polydeoxyribonucleotides (PDRN) derived from purified DNA of salmon or trout gonads, offers a promising approach to antiaging [4, 5, 6]. These products, known for their safety and efficacy, have become widely prescribed in skin rejuvenation [5]. However, while their use is growing, a comprehensive review of their benefits, risks, and applications is lacking.

This systematic review aimed to fill that gap by critically analyzing the existing literature on polynucleotide‐based skin rejuvenation, with a particular focus on their effectiveness and safety profile. Previous publications, including a key consensus by Cavallini et al. [7], have highlighted the potential of PN for skin priming and revitalization, yet further synthesis is needed to consolidate these findings and provide clarity on their clinical utility. By synthesizing data from existing primary studies, this review intends to offer a rigorous evaluation of PN treatments.

## 2 Materials and Methods

### 2.1 Search Strategy

A systematic review of three databases, Embase (Ovid), MEDLINE (Ovid), and Cochrane Library, was performed according to Preferred Reporting Items for Systematic Reviews and Meta‐Analyses (PRISMA) guidelines [8] using the following search terms: “polynucleotide,” “polydeoxyribonucleotide,” “PN‐HPTTM,” “aesthetic medicine,” “hair restoration,” “skin rejuvenation,” and “facial aesthetic.” We also searched clinical trials registers (ClinicalTrials.gov), using the following search terms: “polynucleotides,” and “polydeoxyribonucleotide.” Boolean operators were used to combine search concepts and subject headings (e.g., “AND”; “OR”). A limit was applied to English language and studies published between January 01, 2010, and January 01, 2024. We also searched the reference lists of included studies and other papers related to the topic of interest to identify potential studies that the original electronic database search missed. The search was repeated before the final analysis to ensure that no new studies would be missed from the final synthesis. The protocol for this study was registered on PROSPERO (CRD42024588712).

### 2.2 Eligibility Criteria

Eligibility criteria are summarized in Table 1.

**TABLE 1:** Selection criteria.

	Inclusion criteria	Exclusion criteria
Participants	Healthy adults (≥ 18 years) of any age/sex/ethnicity undergoing PN treatment for skin or hair restoration	Patients ≤ 17 years old Animal studies In vitro studies
Intervention	PN injections	Combination treatments of PN and another antiaging treatment
Comparison	Before/after PN use No use of PN	
Outcomes	Evaluation of the molecular process and influence of polynucleotides on the anatomy and physiology of different skin layers	
Study design	Primary research including RCTs and case studies	Abstracts, systematic reviews, meta‐analyses, editorials, letters

### 2.3 Study Selection

Two independent reviewers (S.L., and G.C.), with academic/research and esthetic backgrounds, screened records for inclusion. References were imported into the Covidence software, where duplicates were excluded. Titles and abstracts were screened against criteria by two reviewers. Eligible studies' full texts were retrieved, reviewed, and qualified for inclusion by two independent reviewers. Disagreements were resolved through discussion; a third reviewer was consulted if needed.

### 2.4 Data Extraction and Synthesis

Two authors (S.L. and G.C.) independently performed data extraction for each paper. Data were recorded using a predefined template in Covidence. Extracted information included author(s), publication year, country, study aim(s), design, sample size, participant characteristics, cosmetic indication, treatment details, adverse events, and key findings.

Given the study heterogeneity, a narrative synthesis was used, involving tabulation, textual descriptions, grouping, and content analysis [9]. One author (S.L.) initiated synthesis, with iterative discussions refining the review's essential elements with other authors.

### 2.5 Quality Assessment

The quality of the included papers was evaluated using the Critical Appraisal Skills Program (CASP) tool for Randomized Control Trials and Case Studies [10]. The quality assessment was conducted by two independent reviewers (S.L. and S.B.) and any discrepancies were resolved by a third author (G.C.).

## 3 Results

**FIGURE 1:**
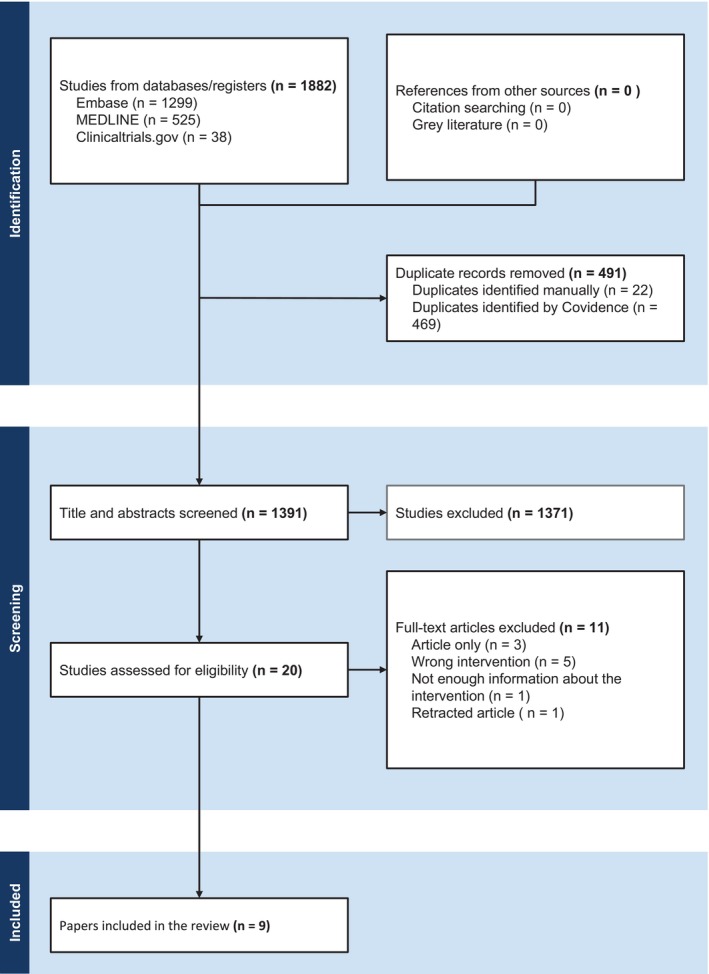
PRISMA flow chart.

A total of 1391 studies were identified through the search, of which 491 were duplicates. At full‐text screening, a potentially eligible study was excluded as the publication has been retracked and we could not identify a newer version of this [11]. After screening was completed, nine studies evaluating the effectiveness of polynucleotides in esthetic medicine were included in this review [12, 13, 14, 15, 16, 17, 18, 19, 20] (See Figure 1).

### 3.1 Methodological Quality

The included studies varied based on their quality (See Tables S1 and S2 in Appendix S1), with some items in the quality assessment tool not reported across studies. Based on the critical appraisal tools, none of the included studies achieved full scores in the quality appraisal, and these were not excluded based on poor quality. Variations in settings and outcomes precluded meta‐analysis.

### 3.2 Study Characteristics

The studies were published between 2014 and 2022. Most (7/9) studies took place in Korea [12, 13, 14, 15, 16, 19, 20], apart from two studies taking place in Italy [17, 18]. The nine studies represent a total sample size of 219 patients (range 5–72), with a mean age ranging between 33.9 and 55.71. Most studies had exclusively female patients [13, 14, 15, 17, 18, 19]. The study characteristics are presented in Table 2.

**TABLE 2:** Study and sample characteristics.

Author (year), country, study design	Population	Procedural characteristics	Intervention group	Control group	Number of treatments	Follow‐up	Complications
Pak et al. (2014) [12] Korea RCT	*n* = 72 Mean age: 40–50 year Sex: 68 female, 4 male Ethnicity: Korean	Indication/area: crow's feet Dose: 1.0 mL	Rejuran (PN 20 mg/mL)	Yvoire‐Hydro (LG Life Sciences, Seoul, Korea), filled with high‐concentration, non‐cross‐linked hyaluronic acid, and DPBS (Dulbecco's phosphate‐buffered saline, Join Bio‐Innovation, Seoul, Korea)	3 treatments at 2 weeks' intervals	Duration: 12 weeks Time points: Total of four injections, every 2 weeks 2, 4, 6, 8, 10, 18 weeks	Treatment‐emergent local AEs occurred at 31.9% of participants. Pneumonia and ligament disorder occurred in two subjects. No causal relationship with the treatment
Lee et al. (2015) [13] Korea RCT	*n* = 40 (20 patients in intervention and 20 in control group) Mean age: 33.9 Sex: 100% female Ethnicity: Korean	Indication/area: female pattern hair loss, hair, along the frontal, mid, and vertex areas Dose: 2.0 mL Technique: needle	PRP & PN	PDRN (Placentex Integro; Mastelli Ltd., Sanremo, Italy)	12 treatments with 1‐week interval	Duration: 13 weeks Time points: 1, 2, 3, 4, 5, 6, 7, 8, 9, 10, 11, 12, 13 weeks	Tolerable pain during the procedures (*n* = 12; 60%), mild itching sensations, and desquamation thereafter (*n* = 1; 10%)
Noh et al. (2016) [14] Korea Case study	*n* = 6 Mean age: 45.2 ± 12.0 Sex: 100% female Ethnicity: Korean	Indication/area: postinflammatory facial hyperpigmentation Dose: 1.0 mL/0.05–0.1 mL per injection point Technique: injections 1 cm apart	Placentex PDRN (one vial contains 5.625 mg PDRN in 3 mL)	N/A	3 treatments with 4 weeks' intervals	Duration: 12 weeks Time points: 4, 8, and 12 weeks	Not reported
Park et al. (2016) [15] Korea Case study	*n* = 5 Mean age: 36 Sex: 100% Female Ethnicity: Korean	Indication/area: Cheeks Dose: 1.0 mL/0.05 mL per cheek Technique: intradermal, serial puncture technique, 40 injections with 1 cm apart, 33G needle	PN 20 mg/mL	N/A	4 treatments at 2‐week intervals	Duration: 12 weeks Time points: 2, 4 and 12 weeks	Mild pain during treatment and focal bruising
Jeong et al. (2019) [16] Korea RCT	*n* = 29 Mean age: 20 Sex: not reported Ethnicity: Korean	Indication/area: Crow's feet Dose: 1.0 mL Technique: linear threads, 33G needle	Polycaprolactone	PN 20 mg/mL	4 treatments with 2 week intervals	Duration: 12 weeks Time points: 4, 12 weeks	Site edema (50.00%), injection site pain (23.33%), pruritus (13.33%), erythema (10.00%)
Araco and Araco (2021) [17] Italy Double‐blinded RCT	*n* = 20 (10 patients in intervention & 10 in control group) Mean age: 36.3 ± 5.48 (37.4 ± 3.29 in the PN group) Sex: 100% female Ethnicity: Italian	Indication/area: Acne scars Dose: average 4.0 mL Technique: subdermal, 30 microdrops (0.1–0.2 mL at each injection point), 30 g, 13 mm needle	PN	Saline	2 treatments with 3 weeks intervals	Duration: 12 weeks (3 months) Time points: 1 and 3 months	No minor or major side effects were reported during the study
Araco, Araco, Raichi (2022) [18] Italy Prospective, randomized, exploratory study	*n* = 20 Mean age: 49.7 ± 5.82 years Sex: 100% female Ethnicity: Italian	Indication/area: Nasolabial folds Dose: 4.0 mL Technique: intradermally, 10 microdrops (0.2 mL at each injection point), 30G, 8 mm needle	PN Saline	Saline	3 treatments with 3 weeks intervals	Duration: 6 months Time points: 6 weeks, 3 months, and 6 months	No side effects experienced
Lee et al. (2022) [19] Korea Randomized, double‐blind, split‐face trial	*n* = 27 Mean age: 48.78 Sex: 100% female Ethnicity: Korean	Indication/area: Periocular (crow's feet and infraorbital) Dose: 1.0 mL Technique: injections	Rejuran PN 20 mg/mL	Yvoire‐Hydro HA	3 treatments with 2 weeks intervals	Duration: 28 weeks Time points: 2, 4, 10, 16, and 28 weeks	Erythema (3.7%), pruritus (3.7%), transient irritation (3.7%)
Kim et al. (2022) [20] Korea Case study	*n* = 30 Mean age: 55.71 Sex: 27 female and 3 male Ethnicity: Korean	Indication/area: crow's feet Dose: 1.0 mL Injection technique: 34G needle, serial puncture techniques, 0.5 mL of the filler was injected at the corner of each eye, and the dose was altered based on the investigator's discretion	PN 20 mg/mL	N/A	4 treatments with 2 weeks' intervals	Duration: 18 weeks Time points: 2, 4, 6, 8, 10, and 18 weeks	Transient edema in the injection site (71.42%), erythema (10.71%) and itching (3.57%)

### 3.3 Procedural Characteristics

Procedural characteristics varied widely, with crow's feet being the predominant treatment area in four studies [12, 16, 19, 20] (Table 2). Other areas included the scalp for female hair loss [13], facial acne scars [16], nasolabial folds [18], postinflammatory facial hyperpigmentation areas [14], and cheeks [15]. Injection techniques primarily involved serial injections spaced approximately 1 cm apart [13, 15, 18, 19, 20], although variations such as linear threads [16] or subdermal injections [16] were noted. Needle sizes ranged from 30G [17, 18], 33G [13, 14], and 34G [20]. Product quantities varied, with studies administering 0.5 mL [14, 20] to 1 mL [16, 19] per side, while others used 2 mL [13, 18] or 3–6 mL [17] depending on the treatment area and protocol. Two studies allowed dose adjustments, but none reported protocol deviations [12, 20]. A variation was also present regarding treatment intervals, with most studies incorporating a 4‐week interval (Table 3).

**TABLE 3:** Time points for injections/assessment/follow‐up.

Author (year)	Preprocedure	2 weeks	4 weeks	6 weeks	8 weeks	10 weeks	12 weeks	16 weeks	18 weeks	24 weeks (6 months)	28 weeks
Pak et al. (2014) [12]	X	X	X	X	X	X			X		
Lee et al. (2015) [13]	X	(Weekly intervals for 12 weeks)									
Noh et al. (2016) [14]	X		X		X		X				
Park et al. (2016) [15]	X	X	X				X				
Jeong et al. (2019) [16]	X		X				X				
Araco and Araco (2021) [17]	X		X				X				
Araco, Araco, Raichi (2022) [18]	X			X			X			X	
Lee et al. (2022) [19]	X	X	X			X		X			X
Kim et al. (2022) [20]	X	X	X	X	X	X			X		

### 3.4 Measurements

Studies employed various methods for assessing treatment effectiveness. Three studies used a combination of digital photography and three‐dimensional (3D) analysis to assess treatment effectiveness [16, 17, 18]. Four studies [17, 18, 19, 20] used Antera 3D for both static and dynamic analysis, evaluating wrinkles, skin texture, pores, depression/sagging, melanin, and hemoglobin. Kim et al.'s [20] study utilized a specific filter for fine wrinkle assessment. Jeong's and Park's [14, 15] studies employed the PRIMOS software for 3D analysis. Araco's studies [17, 18] used a Nikon camera for digital photographs. Three studies [11, 12, 13] relied solely on digital photography without 3D analysis. Lee et al. [13] assessed hair growth using 40‐fold magnification, employing the Follioscope PT software. Noh's study used a 5‐point scale for efficacy based on photographs: (1) little or no improvement (0%–10%); (2) noticeable improvement (10%–25%); (3) fair improvement (25%–50%); (4) good improvement (50%–75%); and (5) excellent improvement (> 75%). Studies assessing treatment effectiveness with photography stressed the need for consistent positioning and lighting to ensure reproducibility in digital images [14, 17, 18].

Park et al.'s study used Visioface (Quick, Courage1Khazaka electronic GmbH, Germany) and Skin scanner (DUB‐USB, Taberna pro medicum, Germany) to evaluate pore size and skin thickness, respectively [15]. Skin tone and melanin images were assessed using VISIA‐CR (2.2 Deluxe, CANFIELD, Germany) and analyzed with Image‐Pro. Moire (MBS‐100, Stradek, Korea) was utilized to evaluate sagging. Studies focusing on PN's impact on crow's feet utilized the crow's feet grading score for clinical efficacy assessment [13, 15, 20]. The same studies also used the global esthetic improvement scale (GAIS) for self‐assessment to evaluate participant satisfaction [16, 19, 20].

### 3.5 Outcomes

#### 3.5.1 Wrinkle Reduction

Six studies examined wrinkle reduction after PN injections [12, 15, 16, 17, 18, 20], with all reporting improvements, though only half showed statistically significant results. Araco and Araco [17] found notable wrinkle reduction at 3 months (29.24 ± 1.1) compared with baseline (33.14 ± 1.33), *p* < 0.05. His 2022 study also revealed consistent improvement at 6 weeks (27.6 ± 2.47), 3 months (24.0 ± 1.00), and 6 months (26.5 ± 1.10), significantly lower than baseline (36.1 ± 1.76, *p* < 0.05) [18]. Kim et al.'s study reported significant reductions at 8 (16.02 ± 5.78), 10 (15.72 ± 5.52), and 18 weeks (17.21 ± 5.69) versus baseline (19.39 ± 7.84), *p* < 0.001 [20]. Jeong et al.'s study noted improvements in resting wrinkle severity but lacked statistical significance [16]. Pak et al. [12] observed a 95.7% improvement in crow's feet 12 weeks post treatment. Another study found women in their 40s showed better results within 2 weeks compared to 12 weeks post treatment [15].

#### 3.5.2 Skin Texture and Elasticity

Regarding skin texture, more visible results were reported within 3 months of the treatment. In Park et al.'s study [15], women in their 30s experienced a more visible improvement regarding skin thickness and pore reduction at 12‐week follow‐up compared to 2 weeks. However, women in the 40s had a more even skin tone during the first 2 weeks post treatment compared with 12 weeks [15]. Araco and Araco's study reported that skin texture levels between 1 and 3 months follow‐up are not too dissimilar (27.79 ± 0.74 vs. 2.75 ± 0.54) [17]. However, these are significantly better than baseline (37.1 ± 0.69) (*p* < 0.005) [17]. Another study highlighted that at 3‐month follow up the biggest improvement was noticed in skin texture compared with baseline (16.1 ± 2.19 vs. 29.1 ± 0.50) (*p* < 0.05) [16]. Although at 6‐month follow‐up, skin texture improvement seems to decline (18.9 ± 1.41), and it did not return to the pre‐treatment levels (29.1 ± 0.50) (*p* < 0.05) [16].

A significant improvement in skin texture can be seen up to 18 weeks (17.91 ± 6.53) post treatment compared with baseline (20.70 ± 9.15) (*p* < 0.001) [20]. Similar improvements were also presented at 8 (16.59 ± 6.76) and 10 weeks (16.23 ± 6.24) (*p* < 0.001) [19]. In Lee et al.'s study, the improvement rate of pore volume in the PN group was higher than that in the HA group at 16 (*p* = 0.0091) and 28 weeks (*p* = 0.0045) [19]. Correlation analysis revealed a positive relationship between the improvement rates of roughness and pore volume at Week 10 (*r* = 0.60698, *p* < 0.0001), Week 16 (*r* = 0.60448, *p* < 0.0001), and Week 28 (*r* = 0.80569, *p* < 0.0001). Additionally, Lee et al.'s study indicated that a significant increase was observed in the level of hydration in the PN compared to the HA group (*p* = 0.0486) at 16 weeks [19].

One study reported that sagging was reduced 8 (0.91 ± 0.48), 10 (0.88 ± 0.47), and 18 (1.01 ± 0.47) weeks compared WITH baseline (1.19 ± 0.65) [20]. However, only the 18 weeks results were statistically significant (*p* < 0.001) [20]. On the other hand, in Park et al.'s study [15], women in their 40s exhibited improvement in sagging within the initial 2 weeks after treatment, contrasting with the results at the 12‐week follow‐up.

#### 3.5.3 Hemoglobin and Melanin

Five studies [14, 15, 17, 18, 20] addressed changes in hemoglobin and melanin levels. Araco and Araco's study on acne scars showed a nonsignificant hemoglobin increase [17]. In Araco, Araco, Raichi's study on nasolabial folds, a significant hemoglobin increase was observed at 3 months (152.0 ± 13.20, *p* < 0.05) and 6 months (160.0 ± 12.20, p < 0.05), but not at 6 weeks (137.7 ± 12.00) [18]. Kim et al.'s (2022) study [20] presented an initial increase at 8 weeks (0.93 ± 0.17) compared with baseline (0.89 ± 0.14) (*p* = 0.018), declining at 10 weeks (0.90 ± 0.16, *p* = 0.359) and 18 weeks (0.89 ± 0.17, *p* = 0.015) follow‐up, with only the latter being statistically significant.

Regarding melanin levels, Park et al.'s 2016 study suggested better improvement in the first 2 weeks for women in their 40s, though statistical significance was not reported [15]. Kim et al.'s 2022 study reported an initial reduction at 8 weeks (0.60 ± 0.08, *p* = 0.022) and 10 weeks follow‐up (0.59 ± 0.07, *p* = 0.002) compared with baseline (0.61 ± 0.08), but no further decline at 18 weeks (0.61 ± 0.07, *p* = 0.968), though not statistically significant [20]. Noh et al.'s study showed postinflammatory pigmentation improvement after three PN sessions, with all patients exhibiting significant improvement by 12 weeks [14].

### 3.6 Adverse Events

None of the included studies reported severe adverse events. Two studies reported no post‐treatment side effects [17, 18], while others noted mild ones like localized pain [13, 15, 16], site edema [16, 20], pruritus [14, 16, 20], bruising [15], erythema [13, 16, 20], and transient irritation (3.7%) [13] typically resolving within a week. Similar side effects were observed in control groups in one study [16].

### 3.7 Patient Satisfaction

Four studies [12, 13, 14, 15, 16] did not assess satisfaction. Park et al. reported all participants were satisfied and willing to undergo PN treatment again [15]. Two studies used a patient satisfaction questionnaire (PSQ) developed by the study team, measuring satisfaction on a scale of 0 to 10 [17, 18] showing moderate‐to‐high satisfaction levels in acne scarring [17] and nasolabial folds [18] studies. Three studies [16, 19, 20] used the GAIS for self‐assessment, with Kim's study [20] indicating higher self‐assessment scores than investigator assessment.

## 4 Discussion

The nine studies included in this review touched on promising results of PN use in a plethora of therapeutic areas through the stimulation of cell growth and tissue repair. However, given the low and moderate quality of existing studies, further, high‐quality research is needed to draw firm conclusions.

During the last few years, there is a shift in esthetic medicine toward subtle and natural skin rejuvenation. In this environment, Cavallini et al. highlight the growing dermatologist acceptance of PNs in addressing fine lines and wrinkles [7]. Our findings support a multilevel and long‐lasting beneficial effect in skin status that is much superior to simple skin boosters that only offer immediate hydration and glow [21]. In comparison with well‐established blood‐derived agents such as Platelet Rich Plasma (PRP) and Platelet‐Rich Fibrin (PRF) that can also lead to a natural regenerative result, PNs seem as a time‐saving, cheaper, and less clinician‐dependent treatment modality [22].

An important advantageous feature of PN use that is reiterated by our study is the safety profile that omits any serious complication. In the contrary, HA fillers and collagen‐stimulating agents can potentially lead to a wide range of adverse events from a disfiguring overcorrection to side effects that are difficult to manage, such as granulomas and long‐lasting edema, or even devastating ones such as skin necrosis and vision loss [23]. Despite promising results, further assessment of PNs in esthetics is crucial. Standardized study characteristics and methodologies, along with larger populations and longer follow‐up periods, are essential. Such research will optimize treatment efficacy and patient satisfaction, unlocking PNs' full potential in clinical practice.

### 4.1 Limitations

This review has several limitations. The inclusion of only nine studies may limit the evidence's generalizability. Variations in study design, data collection, sample size, and outcome measures made synthesis challenging. The narrative synthesis approach introduces subjectivity despite efforts to mitigate it. Studies varied in quality, with none achieving full scores in critical appraisal. Hence, the results need to be reviewed with caution. Despite a thorough literature search, the exclusion of non‐English studies may have led to missed relevant research.

## 5 Conclusions

Polynucleotides offer a natural, effective, and safe option for skin rejuvenation. Their capacity to promote collagen production, tissue repair, and improve skin quality and health aligns notably with the industry's shift toward more regenerative, safer and less invasive treatments. Although further research is necessary to outline standardized treatment protocols, the consensus of the current literature poses PNs as a valuable addition to the repertoire of any esthetic clinician, potentially transcending other more traditional treatments in the improvement of skin appearance.

## Author Contributions

**Smaragda Lampridou:** methodology, screening and data extraction, data analysis, writing – original draft. **Sian Bassett:** screening and data extraction, writing – review and editing. **Maurizio Cavallini:** writing – review and editing, supervision. **George Christopoulos:** conceptualization, methodology, screening and data extraction, writing – review and editing, supervision.

## Ethics Statement

The authors have nothing to report.

## Conflicts of Interest

S.L. has no conflicts of interest to declare. S.B. is the owner of Sian Aesthetics. M.C. is a Research and Development (R&D) steering board member and a tutor in continuous medical education activities for Mastelli Srl (Sanremo, Italy). M.C. is employed by Centro Diagnostico Italiano. G.C. is employed by the Ghanem Clinic and he is a Key Opinion Leader (KOL) and a clinical trainer for Dermafocus Ltd., a distributing company of polynucleotides (PN‐HPT) medical devices from Mastelli Srl (Sanremo, Italy). Dermafocus Ltd. or Mastelli Srl were not involved in this research and no grants were received.

## Supporting information


Appendix S1.

## Data Availability

All data are incorporated into the article and its Supporting Information.
