# Supplementary material for: The Effectiveness of Polynucleotides in Esthetic Medicine: A Systematic Review
Source: J Cosmet Dermatol. 2024 Dec 8;24(2):e16721. doi: 10.1111/jocd.16721 (PMC11845969; doi:10.1111/jocd.16721)
Supplement: Supplementary file 1 — Appendix S1. [file JOCD-24-e16721-s001.docx]

**APPENDIX**

Table 1. CASP Checklist for Case-Control Studies.

|  | Noh et al., 2016 [13] | Park et al, 2016 [14] | Kim et al., 2022 [19] |
| --- | --- | --- | --- |
| **Section A: Are the results of the study valid?** | | | |
| Did the study address a clearly focused issue? | Yes | Yes | Yes |
| Did the authors use an appropriate method to answer their question? | Can’t tell | Can’t tell | Yes |
| Were the cases recruited in an acceptable way? | Can’t tell | No | Can’ tell |
| Were the controls recruited in an acceptable way? | Can't tell | No | Can’t tell |
| Was the exposure accurately measured to minimise bias? | Can't tell | Can't tell | Yes |
| Aside from the experimental intervention, were the groups treated equally? | Yes | Yes | Yes |
| Have the authors taken account of the potential confounding factors in the design and/or in their analysis? | Can’t tell | No | No |
| **Section B: What are the results?** | | | |
| Do you believe the results? | Can’t tell | No | Can’t tell |
| **Section C: Will the results help locally?** | | | |
| Can the results be applied to the local population? | Can’t tell | Can't tell | Can’t tell |
| Do the results of this study fit with other available evidence? | Can't tell | Can't tell | Yes |

Table 2. CASP Checklist for Randomised Controlled Trials.

|  | Pak et al., 2014 [11] | Lee et al., 2015 [12] | Jeong et al., 2019 [15] | Araco et al., 2021 [16] | Araco et al., 2022 [17] | Lee et al., 2022 [18] |
| --- | --- | --- | --- | --- | --- | --- |
| **Section A: Is the basic study design valid for a randomised controlled trial?** | | | | | | |
| Did the study address a clearly focused research question? | Yes | Yes | Yes | Yes | Yes | Yes |
| Was the assignment of participants to interventions randomised? | Yes | Can't tell | Yes | Yes | Can’t Tell | Yes |
| Were all participants who entered the study accounted for at its conclusion? | Yes | Yes | Yes | Yes | Yes | Yes |
| **Section B: Was the study methodologically sound?** | | | | | | |
| Were the investigators “blind” to intervention they were giving to participants? | Can't tell | Can't tell | Yes | Yes | Can't tell | Yes |
| Were the people assessing/analysing outcome/s “blinded”? | Yes | Can’t tell | No | No | No | Yes |
| Have the authors taken account of the potential confounding factors in the design and/or in their analysis? | No | Can't tell | Yes | Can't tell | Can't tell | Yes |
| Were the study groups similar at the start if the randomised controlled trial? | Yes | Yes | Yes | Yes | Yes | Yes |
| Apart from the experimental intervention, did each study group receive the same level of care (that is, were they treated equally)? | Yes | Yes | Yes | Yes | Yes | Yes |
| **Section C: What are the results?** | | | | | | |
| Were the effects of intervention reported comprehensively? | Yes | Yes | Can't tell | Yes | Yes | Yes |
| Was the precision of the estimate of the intervention or treatment effect reported? | Yes | No | No | No | No | No |
| Do the benefits of the experimental intervention outweigh the harms and costs? | Can't tell | Can't tell | Can't tell | Can't tell | Can't tell | Can't tell |
| **Section D: Will the results help locally?** | | | | | | |
| Can the results be applied to your local population/in your context? | Yes | Can’t tell | Can’t tell | Yes | Yes | Yes |
| Would the experimental intervention provide greater value to the people in your care than any of the existing interventions? | Yes | Can’t tell | Can’t tell | Yes | Yes | Yes |
